# Supplementary figures and images for: Anti-Inflammatory Activities of Euglena gracilis Extracts
Source: Microorganisms. 2021 Sep 29;9(10):2058. doi: 10.3390/microorganisms9102058 (PMC8537577; doi:10.3390/microorganisms9102058)

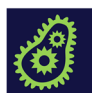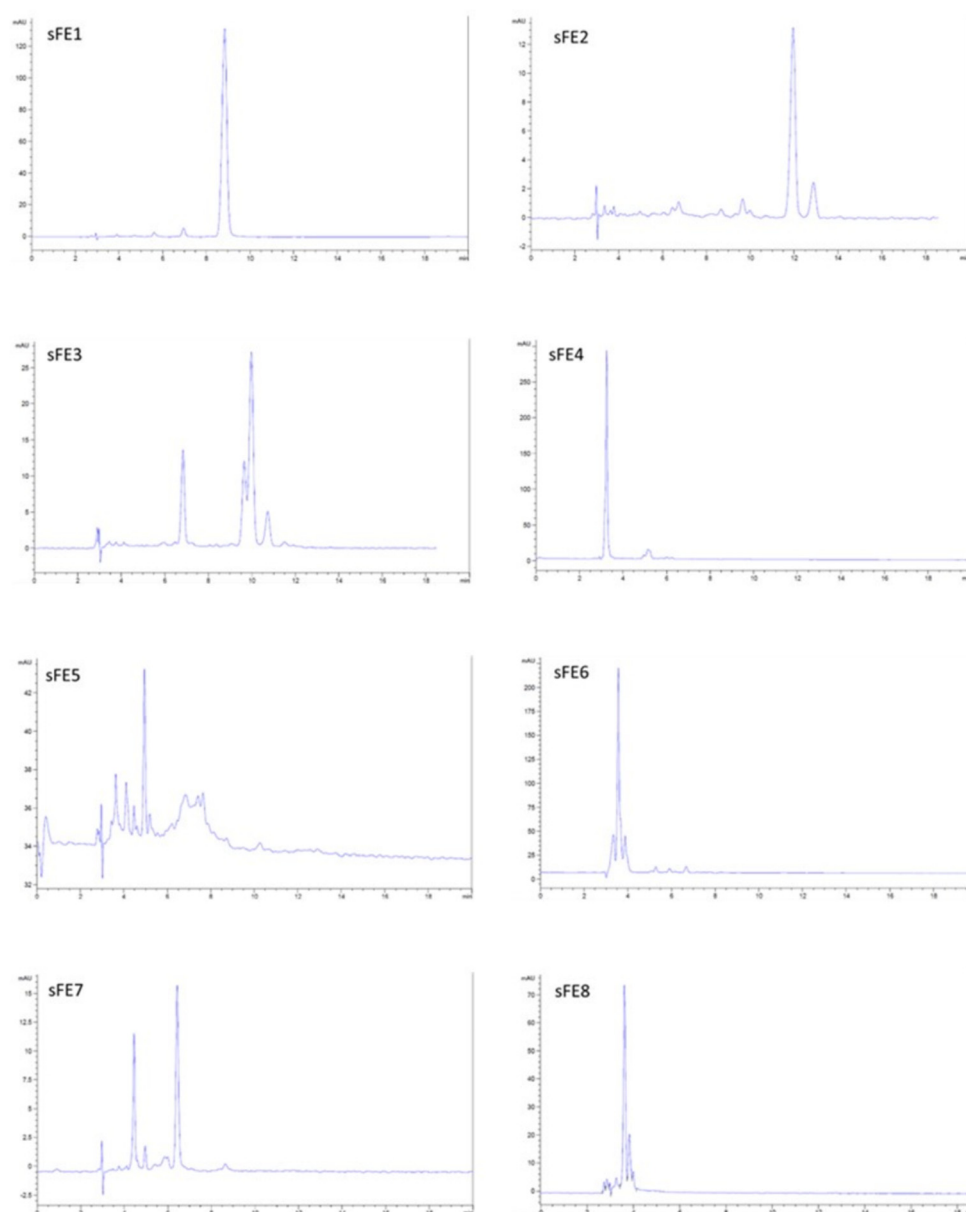

Figure S1. Chromatograms of subfractions sFE1 ÷ sFE8 acquired at 438 nm.

Supplement: Supplementary file 1 [file microorganisms-09-02058-s001.zip › microorganisms-1367687-supplementary.pdf]
